# Influence of Surface Modified Nanodiamonds on Dielectric and Mechanical Properties of Silicone Composites

**DOI:** 10.3390/polym11071104

**Published:** 2019-06-29

**Authors:** Alexandra Shakun, Rafal Anyszka, Essi Sarlin, Anke Blume, Jyrki Vuorinen

**Affiliations:** 1 Materials Science and Environmental Engineering, Tampere University, P.O. Box 589, FI-33014 Tampere, Finland; 2 Elastomer Technology and Engineering, University of Twente, P.O. Box 217, 7500 AE Enschede, The Netherlands; 3 Institute of Polymer and Dye Technology, Lodz University of Technology, Stefanowskiego 12/16, 90-924 Lodz, Poland

**Keywords:** nanodiamonds, surface modification, silicone, dielectric spectroscopy

## Abstract

Detonation nanodiamonds, also known as ultradispersed diamonds, possess versatile chemically active surfaces, which can be adjusted to improve their interaction with elastomers. Such improvements can result in decreased dielectric and viscous losses of the composites without compromising other in-rubber properties, thus making the composites suitable for new demanding applications, such as energy harvesting. However, in most cases, surface modification of nanodiamonds requires the use of strong chemicals and high temperatures. The present study offers a less time-consuming functionalization method at 40 °C via reaction between the epoxy-rings of the modifier and carboxylic groups at the nanodiamond surface. This allows decorating the nanodiamond surface with chemical groups that are able to participate in the crosslinking reaction, thus creating strong interaction between filler and elastomer. Addition of 0.1 phr (parts per hundred rubber) of modified nanodiamonds into the silicone matrix results in about fivefold decreased electric losses at 1 Hz due to a reduced conductivity. Moreover, the mechanical hysteresis loss is reduced more than 50% and dynamic loss tangent at ambient temperature is lowered. Therefore, such materials are recommended for the dielectric energy harvesting application, and they are expected to increase its efficiency.

## 1. Introduction

Detonation-produced nanodiamonds (NDs), also named ultradispersed diamonds (UDDs), are known for their superior mechanical properties, low electrical conductivity, high thermal conductivity and a highly reactive surface. Single ND particles are about 5 nm in diameter consisting of a sp^3^-carbon core, less than 1 nm thick sp^2^–carbon transitional layer and an active surface [1]. The surface of a pristine ND is hydrophilic and mostly contains carboxyls, hydroxyls, lactones, ketones and ethers, but it can be modified and homogenized through a large number of methods [2]. However, due to the presence of carboxyl- and hydroxyl- groups, NDs form strong aggregates and agglomerates, which are difficult to break down, thus affecting the dispersability of NDs. Further surface modification allows introducing complex moieties onto the ND surface and could decrease interactions between the ND particles.

NDs are mostly applied for biomedical, electronic, and tribology applications and in different polymer-based composites [3,4,5]. Moreover, NDs have shown an ability to reduce dielectric losses in some elastomeric matrices [6,7], which can be used for the dielectric energy harvesting. Dielectric energy harvesters can provide sustainable energy from the ambient sources, such as ocean waves [8,9,10,11,12] and human motion [13,14], but their efficiency and economic profitability is dependent, among others, on the material-related losses of the elastomeric membrane. Silicone rubber is often used for dielectric energy harvesting, as it possesses low dielectric losses, low mechanical hysteresis, dynamic damping and stress relaxation [15,16]. However, a modification of the material is needed in order to increase the economic profitability of elastomer-based energy harvesters [17]. A modification of the silicone matrix by covalent attachment of various molecules can not only improve mechanical properties of the material, but also result in lower dielectric losses and better electrical breakdown resistance in dielectric elastomer transducers [16]. Therefore, it is expected that the introduction of NDs into a silicone matrix improves the physical and chemical interactions within the matrix. Furthermore, the introduction of surface modified ND particles is expected to reduce the dielectric and mechanical losses of the composite further and increase its potential in energy harvesting. 

Similar to other nanomaterials, the fabrication of ND-elastomer composites is associated with some challenges. They can be related to the poor ND-elastomer interactions and the strong agglomeration of NDs, resulting in poor dispersion and reduced composite strength. Due to the high surface energy of NDs, breaking up ND aggregates into primary particles cannot be achieved by direct ultrasound processing [18]. Moreover, a direct mixing of the ND powder into the polymer results in filler agglomeration and inadequate dispersion [19,20,21]. The situation can be improved by surface functionalization of NDs. A treatment with silanes creates a hydrophobic ND surface and, therefore, improves the dispersion of NDs in nonpolar organic media. If a vinyl-containing silane is used, it is reported that the dispersion is improved both in nonpolar and polar organic media [22]. However, an increase in the agglomerate size can be observed after silanization due to the condensation reaction between treated particles [23] or a self-condensation of the silane [24]. Owing to the self-condensation reaction, a siloxane shell is formed around the ND particle, which can further bond particles into very large aggregates. Basic conditions and elevated temperature facilitate the condensation of silanol groups [25]. A minimum condensation rate for tri-, di- and monosilanols can be achieved at pH 4, pH 6 and pH 6.5–7, respectively, which allows minimizing the siloxane shell formation [26].

Although NDs are considered easily reactive compared to other carbon materials, their surface modification often requires several steps, strong chemicals and/or high temperatures [27]. For example, the traditional route of ND functionalization often includes a reduction of a pristine ND surface to hydroxyl-groups followed by introduction of amine groups, e.g., by silanization, which is followed by further modifications [28,29]. Thus, more simple and low temperature surface modification methods designed for improved interaction with hydrophobic polymeric matrices are in demand.

A direct functionalization of carboxylated NDs with a vinyl-containing silane has been reported in the work of Hajiali et al. [30], and a possibility of such reaction was mentioned in the latest review of Zhang et al. [3]. However, no strong evidence has been provided for the existence of ND-silane bonds as the result of such modification. The goal of the modification was to create polysiloxane shell on ND surface. A polysiloxane coating is usually achieved through a Stöber process, which involves a silane hydrolysis in basic conditions followed by the reaction between hydrolyzed silane and hydroxyl groups on the ND surface and further polycondensation via creation of Si–O–Si bonds [31]. When ND surface contains –COOH groups instead of –OH groups, a hydrolyzed silane is more likely to react with OH-group of other silane molecules rather than with the –COOH group of the ND. Therefore, a probability of the reaction between carboxylated nanodiamond and hydrolyzed silane needs to be evaluated. Another direct functionalization of carboxylated NDs through the reaction with the epoxy group can be applied [24,32] based on the esterification reaction discussed in the literature [33,34]. However, such modification has been performed on NDs at 140 °C, which is still relatively high temperature.

The aim of the present study was to covalently attach the ND filler to the silicone matrix and promote the interaction between carboxylated NDs and the silicone matrix. Covalent bonding is achieved by decorating NDs with vinyl- groups, which are able to participate in the curing process, and the compatibility with the matrix is improved by introducing alkoxysilanes to the ND surface. Here, two different routes of surface modification of carboxylated NDs are discussed: through silanization with a silane coupling agent and esterification reaction with a modifier containing an epoxy-group. In the study, the probability of ND-silane bond formation is argued in case of carboxylated diamond, but a simple and efficient method of ND surface modification was offered via reaction with an epoxy-group of the modifying agent at 40 °C. Furthermore, the effectiveness of modified NDs in lowering dielectric and viscoelastic losses of silicone composites is discussed. The present study indicates that the application of surface-modified NDs can be beneficial for the dielectric energy harvesting application and could potentially increase the efficiency of dielectric energy generators.

## 2. Materials and Methods

### 2.1. Materials

The study utilized NDs with carboxylated surface (uDiamond Molto Vox, Carbodeon Oy, Vantaa, Finland). According to the supplier, single ND particles are 4.2 nm in size and have an aspect ratio close to 1. According to the particle size distribution measurement conducted by the supplier, agglomerated NDs have a median size of 2.6 μm. The specific surface area of NDs is 330 m^2^/g. More information about the properties of NDs is provided in Table S1. Vinyltrimethoxysilane (referred as VTMS, Sigma Aldrich, St. Louis, MO, USA) as well as 1,2-epoxy-9-decene (referred as ED, abcr GmbH, Karlsruhe, Germany) and 3-glycidoxypropyltrimethoxysilane (referred as GOPTMS, abcr GmbH, Karlsruhe, Germany) were used as surface modifiers. Structures of the used surface modifiers are shown in Figure S1. Glacial acetic acid (Sigma Aldrich) was used for the pH adjustment of the silanization reaction. The composites were prepared using two-component room temperature curable silicone (polydimethylsiloxane—PDMS) with Pt catalyst (Elastosil 601 RT, Wacker Munich, Germany) and 10 phr (parts per hundred rubber) of silicone oil (Rhodorsil 47 V 50, Solvay, Brussels, Belgium).

### 2.2. Nanodiamond Treatment

As-received, carboxylated NDs were oxidized in air at 425 °C for 4 h. Such thermal treatment is expected to increase the amount of surface carboxyl groups available for the further modification by oxidizing the existing oxygen-containing groups, such as lactones. The amount of oxygen-containing surface groups was determined by Boehm titration performed according to the procedure described elsewhere [35]. The total amount of the surface groups, which can be identified by the Boehm titration, was determined by the reaction with NaOH. The number of carboxylic groups—by the reaction with NaHCO_3_, lactones—from the difference between NaOH and NaHCO_3_ tests, and the number of phenolic groups from the difference between Na_2_CO_3_ and NaHCO_3_ reacted with the functional groups of the carbon material

For the ED-based modification, 0.3 g of oxidized NDs were placed into a three-neck flask, dispersed in 80 mL toluene and brought to a constant temperature (40 °C or 100 °C). After temperature stabilization, ED was added slowly in excess molar amount (5:1 based on the amount of surface carboxyl groups on NDs). The reaction was allowed to proceed for 24 h, followed by filtration and washing with toluene. The obtained fillers were further purified in a Soxhlet extractor with toluene for 24 h and dried in an air-ventilated oven at 120 °C for 12 h. The modification of NDs with GOPTMS was performed at 40 °C following the same procedure as for ED.

The VTMS modification was performed as described in [30], with ND to silane mass ratio of 1 to 5, washed with acetone, filtered, purified in Soxhlet extractor with toluene and dried in oven for 24 h at 120 °C. In order to understand the probability of the reaction between the carboxylic groups of ND and the silanol groups of the modifier, additional surface modifications were performed with monoalkoxysilanes (vinyldimethylethoxysilane and octadecyldimethylmethoxysilane) following the same procedure as for VTMS. Monoalkoxysilanes have only one reactive site and cannot form a shell around NDs. Therefore, the presence of these silanes on the ND surface indicates that the reaction between carboxylic- and silanol- groups is possible. When a condensation reaction occurs between two silane molecules, the resulting molecule cannot attach to the ND surface, and it is removed by the following filtration. Vinyldimethylethoxysilane was chosen due to its similarity to VDMS. However, the characteristic FTIR peak of a vinyl- group is often small, and it could remain unnoticed. Therefore, octadecyldimethylmethoxysilane was chosen as the second modifiers due to the distinctive FTIR peak of a long aliphatic chain that could identify the presence of the modifier on the ND surface. The details are provided in the Supplementary Information.

### 2.3. Composite Preparation

For the preparation of ND-PDMS composites, ND powder was dispersed in silicone oil by ultrasonication (FinnSonic m03) for 30 min at room temperature. NDs were used in 0.1 or 1 phr amounts. ND-containing oil was then added to the part A of a two-component silicone containing the catalyst and stirred for 5 min, then combined with part B silicone containing the crosslinker and stirred for another 2 min. The material was poured into molds to form both 1 mm and 0.5 mm thick sheets, and then degassed in vacuum for 30 min to eliminate air bubbles.

### 2.4. Characterization

Dielectric properties of 0.5 mm thick PDMS samples were measured with a Novocontrol Alpha-A dielectric analyzer at room temperature in a frequency range of 5·10^−2^–10^6^ Hz using gold-plated electrodes (20 mm in diameter). The average value of minimum three specimens was reported for each sample. Between the measurements, the samples were stored in the darkness at room temperature. Dynamic mechanical analysis (DMA), stress relaxation and cyclic loading of the samples were performed with Pyris Diamond DMA (Perkin Elmer, Waltham, MA, USA) using the 1 mm thick samples. DMA samples were measured in the tension mode at 1 Hz with 20 µm displacement amplitude and 3 °C/min heating rate. For the stress relaxation measurement, samples were stretched to 10% and the change in force was recorded. The tensile cyclic loading was performed at ambient conditions at 6 mm/min rate for 10 mm test samples with a maximum elongation at 45 ± 1%. The hysteresis loss was calculated by subtracting the integrated loading and unloading curves and dividing the value by the area under the corresponding loading curves. Determination of moduli (stress at certain percent elongation) was performed on Messphysik Midi 10-20 universal tester equipped with a contact long-travel extensometer (MFE, Mess- & Feinwerktechnik GmbH, Velbert, Germany) at 200 mm/min rate. ISO 37 dumb-bell specimen type 3 with 10 mm test length and about 1 mm thickness were used in the test. The average value of three specimen was reported.

Fourier transform infrared spectra (FTIR) was obtained from the NDs with Bruker Tensor 27 in the attenuated total reflectance (ATR) mode in the range from 500 to 4000 cm^−1^ with a diamond crystal background and 4 cm^−1^ resolution. The ND powders were studied with a scanning electron microscope (SEM, Zeiss ULTRAplus, Oberkochen, Germany) equipped with an energy dispersive X-ray spectroscopy detector (EDS, Oxford Instruments X-MaxN 80, Abingdon, UK). The samples were coated with a thin carbon layer to prevent charging during SEM analysis. In the EDS analysis, the average of two-point measurements were reported. Thermogravimetric (TGA) data was obtained from the NDs with TG 209 F3 Tarsus (Netzsch, Selb, Germany) in nitrogen flow. The samples were kept at 125 °C for 30 min to remove loosely bound water and then heated to 850 °C with 20 K/min rate. The effect of NDs on the crystallinity of the PDMS was studied by means of differential scanning calorimetry (DSC) with DSC 214 Polyma (Netzsch) in N2 flow with 10 K/min heating/cooling rate. The degree of crystallinity was calculated by the DSC Proteus Analysis software based on the equation:

(1) $$\%\;\mathrm{Crystallinity}=\frac{\Delta H_{m}-\Delta H_{c}}{\Delta H_{m}^{{}^{\circ}}}\times 100\%,$$

where *ΔH_m_* is the heat of melting, *ΔH_c_* is the heat of cold crystallization and *ΔH_m_°* is the heat of melting of a 100% crystalline polymer.

The apparent crosslink density (1/Q) of the PDMS was calculated for the 1 × 1 cm samples immersed in toluene in closed vessels for 72 h based on the equation [36]:

(2) $$1/Q=1/\left(\frac{m_{s}\;-{\;m}_{d}}{m_{0}}\right)\frac{F}{100},$$

where *m_s_* and *m_d_* are the weight of the swollen and dried specimen, respectively, *m_o_* is the initial weight of the specimen, and *F* is formula weight in phr. Toluene was renewed every 24 h during the measurement. Three parallel samples were used.

ND powder samples were designated as ND- “surface modifier abbreviation”, for example, ND-VTMS. The PDMS sample without additives was designated as PDMS/0, and PDMS with 10 phr silicone oil - as PDMS_oil/0. In case of a ND filler addition, samples were marked as PDMS/”ND modifier type”_”phr amount”, for example, PDMS_oil/VTMS_1. 

## 3. Results and Discussion

### 3.1. Surface Modification of Nanodiamonds

An additional oxidation in air at 425 °C can be employed in order to increase the number of oxygen-containing groups, especially –COOH, on a ND surface [37,38]. During the treatment, ketones, aldehydes, and esters on the ND surface are converted into carboxylic acids, anhydrides, or cyclic ketones. Moreover, some sp^2^ carbon is removed from the surface giving an access to the formation of oxygen-containing groups. The amount and type of oxygen-containing groups on the ND surface before and after oxidation is shown in Table 1. As the result of the oxidation treatment, the total amount of oxygen-containing groups was virtually unchanged, but the amount of carboxyl groups increased 2.5-fold. Such results are probably achieved by the oxidation of lactone groups and creation of new carboxyl-groups upon removal of few surface sp^2^ carbon layers. Based on the titration results oxidized NDs were selected for further modification. Furthermore, the concentration of surface carboxylic groups was used to calculate the amount of the reagents.

**Figure 1:**
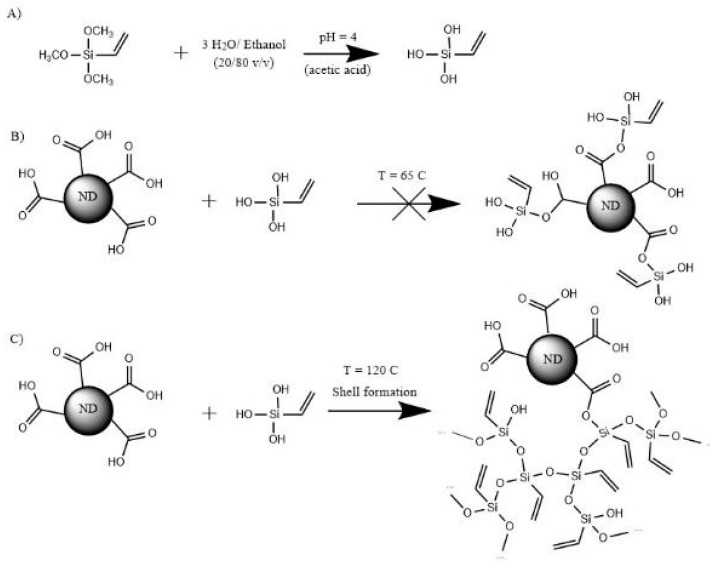
Modification process of carboxylated nanodiamonds (ND) by vinyltrimethoxysilane.

The modification of the ND surface with vinyltrimethoxysilane (VTMS) was performed according to the procedure described in the literature [30]. However, a different mechanism of the reaction is suggested, as shown in Figure 1. During the reaction A, methoxy- groups were hydrolyzed and acetic acid was added to prevent a condensation. The suggested reaction B between the carboxylic and silanol groups was unlikely to proceed, which was confirmed by the use of silanes containing single silanol group, as seen from FTIR spectra and TGA curves depicted in Figures S2 and S3, where no additional peaks appeared. At higher temperature, the shell formation by silanol condensation was proceeded according to the reaction C, but a possibility of the reaction between carboxylic and silanol groups was not fully excluded.

**Figure 2:**
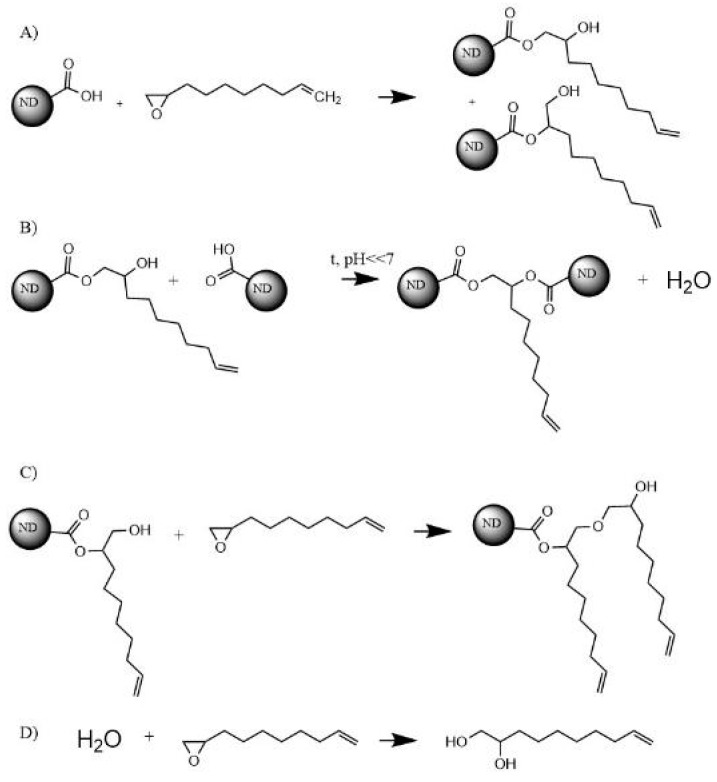
Possible reactions between 1,2-epoxy-9-decene and carboxylated ND (only one carboxyl-group is depicted for clarity).

According to the literature, the reaction between epoxy and carboxyl-group is possible without a catalyst [34]. The probable reactions between carboxylated ND and 1,2-epoxy-9-decene (ED) are shown in Figure 2. Depending on the ring-opening mechanism, two different reaction products can appear. At high temperatures and acidic conditions, the products of the reaction A are prone to bond to ND-COOH via reaction B. In order to maximize the probability of the reaction A between ND-COOH and an epoxy-group, the reaction was performed in toluene at 40 °C and compared to the reaction at 100 °C in terms of the resulting ND surface functionalization. The resulting NDs modified with ED at 40 °C and 100 °C are designated as ND-ED(40C) and ND-ED(100C) respectively. The hydrolyzed epoxy compound and unreacted components were eliminated by filtration and purification stage. It was expected that at 40 °C the reactions A and C occur most probably, while higher temperature would result in more reaction B products and hydrolysis of the epoxy compound as the reaction proceeded. As the excess of ED was used, reaction D was also expected at higher temperature (100 °C).

The reaction between ND and GOPTMS was assumed to follow the same mechanism as for ED, as the direct reaction between carboxyl and silanol was found unlikely, especially at 40 °C (Figure 1, reaction B). Due to the steric effect, type A reaction was expected to be dominant. Moreover, as some water molecules could be present in the system, a condensation of silanol groups could occur upon purification and drying at elevated temperatures.

**Figure 3:**
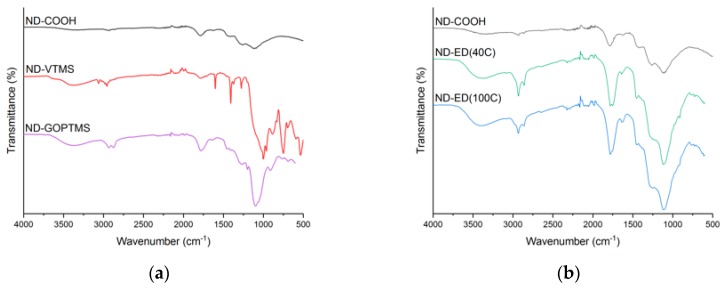
FTIR spectra of NDs modified with: (**a**) silanes; (**b**) 1,2-epoxy-9-decene at different reaction temperatures. Spectra are vertically shifted for clarity.

In the FTIR spectra in Figure 3, oxidized NDs (ND-COOH) show characteristic carbonyl peaks around 1780 cm^−1^ and 1300–900 cm^−1^ as a combination of overlapping peaks from multiple oxygen-containing groups [39]. The same peaks are present in all modified materials, but epoxy-modified materials have more pronounced carbonyl peaks. The presence of the –OH group could be specified at 3100–3600 cm^−1^ (stretching) and 1625 cm^−1^ (bending). All modified fillers have an indication of CH_2_ groups due to peaks at 2860–2930 cm^−1^ and 1408/1453 cm^−1^. 

In the VTMS-modified ND spectrum shown in Figure 3a, a broad peak appeared at 1020 cm^−1^ with a shoulder up to 1110 cm^−1^, corresponding to the overlapping Si–O–Si and Si–C bonds respectively. The appearance of Si-O-Si bonds is a result of the condensation of silanol groups. The intensity of the peaks suggests an extensive shell formation around ND clusters. Peaks at 1275 cm^−1^ corresponds to a Si–C stretching, and the broad peak around 1745 cm^−1^ to the C=O stretch. The broadening at 3200–3400 cm^−1^ confirms the presence of –OH groups and could be an indication of bound water. Peaks at 3062 cm^−1^ and 1601 cm^−1^ appear due to the C=C bond of the vinyl group, and the peak at 750 cm^−1^ – due to the C–H bend of the same group. GOPTMS-modified NDs shown in Figure 3a have distinctive peaks for Si–C bond at 1267 and 1098 cm^−1^ with a broadening towards 1050 cm^−1^, which indicates the presence of Si–O bonds. 

The FTIR-spectra of ED-modified NDs shown in Figure 3b in comparison to ND-COOH. ND-ED(40C) and ND-ED(100C) spectra were almost identical to one another, except the small peak at 3080 cm^−1^ which is related to the vinyl group. This is more pronounced for the ND-ED(40C) than for the ND-ED(100C). Moreover, ND-ED(40C) had double peaks corresponding to the different antisymmetrically coupled C=O stretches (1780 cm^−1^ and 1750 cm^−1^), which could indicate two different reaction A products in epoxy-modification.

**Figure 4:**
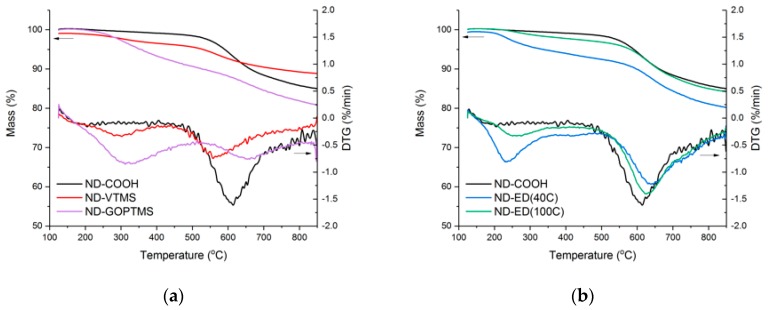
Thermogravimetric (TGA) mass loss and decomposition rate curves of NDs modified with: (**a**) silanes; (**b**) 1,2-epoxy-9-decene, at different reaction temperatures.

According to [39,40], oxidized NDs have shown freely bound water release up to 120 °C and strongly bound water till 200–300 °C, but according to the TGA data shown in Figure 4, ND-COOH experienced no significant mass loss at the mentioned temperatures. Only ND-VTMS and ND-ED(40C) samples show 1% and 0.6% mass loss respectively during the dehydration step indicating the presence of water or solvent residues. When heated above 600 °C in inert atmosphere, unmodified ND first loses its oxygen-containing groups and then undergoes graphitization and sintering above 800 °C resulting in a mass loss up to 10–20% [41], which was observed also in the present study. Among the modified samples, only ND-VTMS has the same onset temperature for the release of strongly bound oxygen-containing groups, while the onset of others shifts to higher temperatures. This finding can indicate that the ND surface was not altered by the VTMS treatment, but a siloxane shell was formed around it.

Unlike ND-COOH, all modified samples show a mass loss in the range of 200–360 °C related to the decomposition of organic groups attached upon modification. The residual mass of a ND-VTMS sample, shown in Figure 4a, is higher than of a ND-COOH sample due to the higher silicon oxide content. Differences in TGA curves of the ED-modified samples are minor, as seen in Figure 4b. The ND-ED(40C) sample seems to have more organic groups attached to the ND, as could be suggested from the proposed reactions A and C (Figure 2). A lower modification temperature and probably more developed surface containing vinyl groups made ND-ED(40C) a good candidate for the further use in PDMS composites. Due to the higher modification temperature and potentially lower vinyl- group content, ND-ED(100C) was not used in PDMS composites.

**Figure 5:**
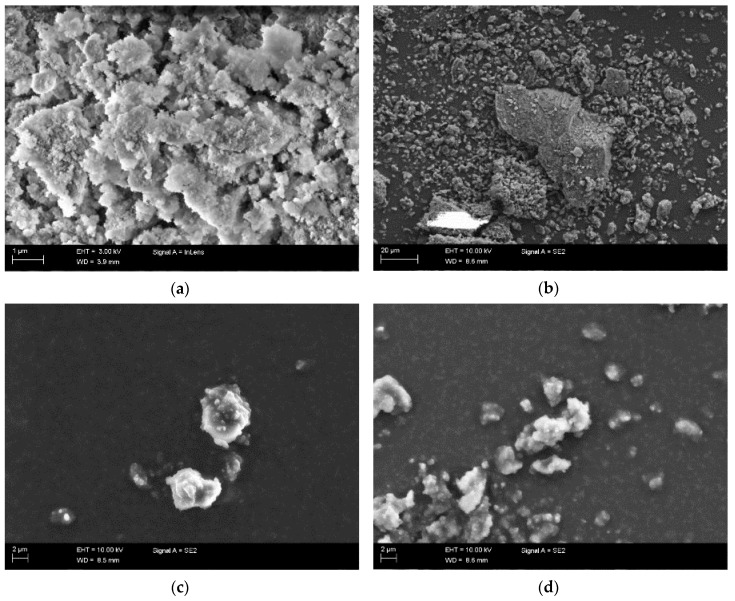
SEM images of ND powders: (**a**) ND-COOH; (**b**) ND-VTMS; (**c**) ND-ED(40C); (**d**) ND-GOPTMS.

The elemental analysis by EDS, presented in Table 2, confirms that the new elements were introduced to the fillers upon modification, and the ratio of the elements was changed. Exceptionally high silicon and oxygen content in ND-VTMS confirmed the extensive shell formation. The presence of a similar shell in the ND-GOPTMS sample is unlikely, as both the Si content and the Si:O ratio was low. The reaction of the epoxy- group with the ND surface is probably much more pronounced and thus the GOPTMS molecule is bounded to the surface and less likely to condense due to restricted mobility. The SEM images of ND powders shown in Figure 5 revealed that all the modified NDs are more agglomerated, and they show a broader range of particle sizes than the initial ND-COOH. Among the modified fillers, ND-VTMS samples have the largest agglomerates, which could be related to the silanol condensation and shell formation around the ND clusters.

### 3.2. Nanodiamond-Silicone Composites

**Figure 6:**
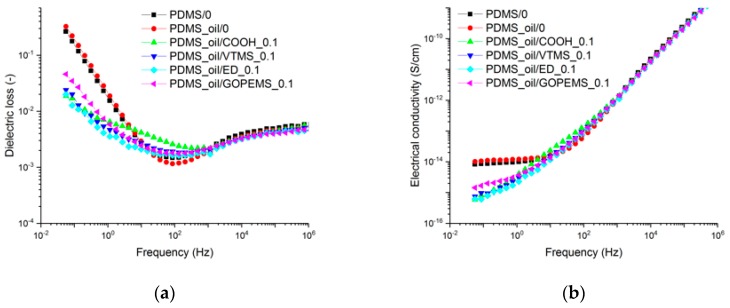
(**a**) Dielectric loss and (**b**) AC electrical conductivity of PDMS samples containing 0.1 phr NDs.

Silicon oil added to the two-component PDMS served as the dispersion media for the NDs. This method helps to avoid common problems with solvent-based dispersion. The effect of oil addition and ND modification on dielectric properties of ND-silicone composites was studied with the focus on dielectric losses. As shown in Figure 6a, no significant difference is found between the non-plasticized PDMS/0 and plasticized PDMS_oil/0. However, the addition of 0.1 phr NDs results in reduced dielectric losses below 10 Hz. For example, the dielectric loss of PDMS_oil/ED_0.1 is 3.6·10^−3^ at 1 Hz, which is about 5 times lower than of PDMS_oil/0. This effect can be related to the reduction of a DC-conductivity effect, which can be seen from the linear slope of the loss curve at low frequency area and from the frequency-independent part of the conductivity curve in Figure 6b. Such losses may be related to the impurities and mobile ions within the matrix that could be trapped and demobilized on the filler surface. For the PDMS_oil/COOH_0.1 sample, an additional broad relaxation peak is visible around a frequency of 10 Hz. Such relaxation was related to the Maxwell-Wagner polarization appearing due to the permittivity and conductivity differences at the matrix-filler interface. As this interfacial relaxation is less pronounced for the modified ND samples, a stronger filler-matrix bond and a more uniform interface is suggested as explanation for this phenomenon. The shape of the curve and positions of the relaxation peaks of PDMS_oil/VTMS_0.1 are very similar to PDMS_oil/ED_0.1, and they lead to decreased dielectric losses four- and fivefold respectively when compared to PDMS_oil/0. Finally, the samples PDMS_oil/COOH, PDMS_oil/ED and PDMS_oil/GOPTMS were selected for further testing due to the differences in their dielectric behavior at the frequencies below 10 Hz.

**Figure 7:**
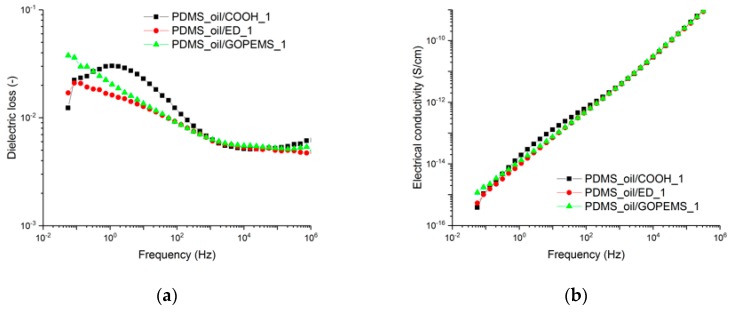
(**a**) Dielectric loss and (**b**) AC electrical conductivity of PDMS samples containing 1 phr NDs.

When the filler amount was increased to 1 phr, an interfacial polarization becomes visible also in PDMS_oil/ED_1 and PDMS_oil/GOPTMS_1 samples (Figure 7b). Nevertheless, it is lower than for the PDMS/COOH_1 sample, which shows a very distinctive broad loss peak around 2 Hz. According to the Figure 7b, the addition of 1 phr of fillers results in a more linear AC conductivity curve, which implies that the reduction of losses is related to the conductivity effect. However, due to the stronger interfacial polarization that increased the losses at low frequencies of the mentioned compounds, it was decided to focus on the 0.1 phr composites, which are also preferable from the economical point of view due to comparably high costs of the nanofillers.

**Figure 8:**
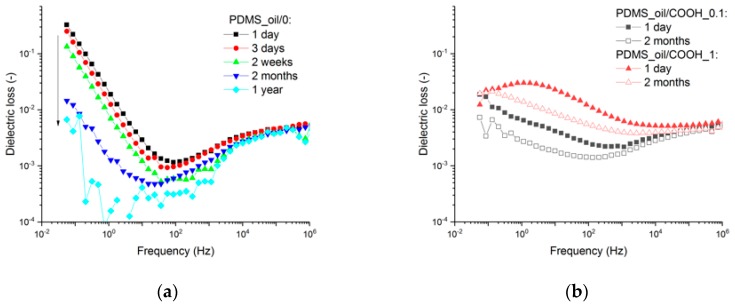
Time-dependent dielectric loss of (**a**) PDMS_ref/0 and (**b**) PDMS with 0.1 and 1 phr of ND-COOH.

Next, as the dielectric loss may change with time [42], the dielectric loss spectra of the samples were measured at different periods of time after curing. According to Figure 8a, the time affects the dielectric loss of PDMS_oil/0 by a small decrease of the relaxation peak around 0.1 MHz and a shift of that peak towards lower frequencies. This relaxation peak is associated with the chain mobility, and therefore, the shift may be explained by a small increase in the degree of crosslinking [43,44]. More significantly, the losses associated with the DC-conductivity are reduced with time, and the largest loss reduction is visible between 1 day and 2 months. This may be related to the progressing crosslinking reaction and migration of ions to the surface of the sample. According to Figure 8b, the reduction in dielectric losses with time is not directly dependent on the filler amount. Moreover, the reduction in losses is less significant than for the reference sample. This suggests that the matrix material is more subjected to time-related changes. Moreover, as the loss properties of the matrix are reduced with time, the positive effect of modified NDs on losses is reduced as well.

From the crosslink density results shown in Table 3, it can be suggested that the crosslinking process is most probably interfered by the presence of methoxysilane or –OH groups at the filler surface. The other ND compounds increased the apparent crosslink density to the level of non-plasticized PDMS, which may suggest certain interaction between the filler and matrix. Although the difference in apparent crosslink densities is notable, moduli change upon the plastification of PDMS and addition of NDs are marginal, as can be seen in Table 3. Due to the combination of low moduli and sufficient crosslink density, PDMS_oil/VTMS_0.1 and PDMS_oil/ED_0.1 have the highest potential for energy harvesting applications compared to the other samples. 

**Figure 9:**
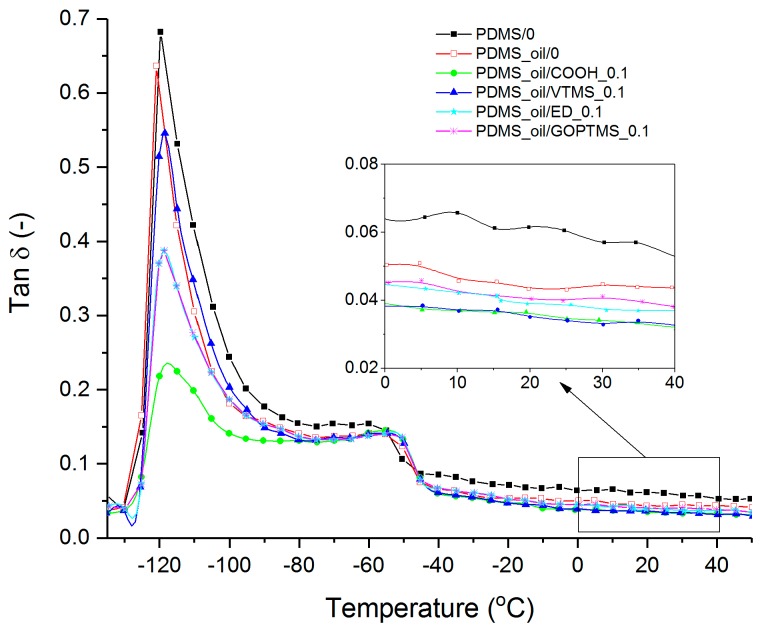
DMA loss tangent curves of PDMS/ND composites as a function of temperature.

DMA is a helpful tool to study the chain dynamics and the damping properties of the materials, where the loss factor (tan δ) indicates the ratio of lost to stored dynamic energy. As seen from Figure 9, the glass transition temperature (Tg) determined at maximum tan δ is lowered upon the addition of plasticizer. The addition of fillers marginally shifts the Tg towards higher temperatures, and also lowers and broadens the transition peaks which is related to the restriction of chain mobility and the differences in the amount of amorphous phase. Additionally, it becomes clear from the insert, tan δ of the ND containing composites is lower than the reference compound indicating less energy loss. PDMS_oil/COOH_0.1 and PDMS_oil/VTMS_0.1 compounds show the lowest tan δ. Such behavior is preferable for the dielectric energy harvesting application, and maybe related to the reduced internal friction as NDs could behave as dry lubricants facilitating the orientation of macromolecules upon the applied stress [7,45]. However, no information is available in the literature concerning the effect of filler-polymer interaction on the dry lubrication action of NDs. 

According to the DSC study (Table 4 and Figure S4), both ND-COOH and modified NDs are expected to affect the orientation of the PDMS molecules. As the addition of 0.1 phr NDs resulted in the increased degree of crystallinity, NDs are expected to act as crystallization centers. Moreover, higher glass transition temperatures of PDMS_oil/VTMS_0.1 and PDMS_oil/ED_0.1 suggests stronger filler-matrix interaction, which can be due to the presence of chemical bonds. The compounds also show no cold crystallization peaks, indicating that the material was fully crystallized during the initial cooling. Although PDMS_oil/COOH_0.1 and PDMS_oil/GOPTMS_0.1 serve as crystallization centers and show similar melting behavior as the previous compounds, the increase in *T*_g_ is not very significant. This can be due to the physical nature of the filler-matrix interactions. Moreover, PDMS_oil/GOPTMS_0.1 has the highest cold crystallization peak and the lowest degree of crystallinity, which can be related to more branched and bulky modifier used for NDs.

The mechanical losses of the samples at higher strains were studied by the cyclic loading and stress relaxation. Due to the low amount of fillers a low Mullins effect was expected. As can be seen from the Table 5, the addition of plasticizer to PDMS reduces the stress losses without notable changes in mechanical hysteresis measured at the 10th cycle. The addition of ND-COOH further reduces the stress loss percentage and hysteresis losses at the first cycle, but again resulted in no significant change after the 10th cycle. All the compounds filled with modified NDs experience a positive hysteresis reduction at the 10th cycle. For example, the hysteresis losses in PDMS_oil/ED_0.1 and PDMS_oil/GOPTMS_0.1 are 53% and 42% lower than the reference. Moreover, the stress loss after 1 and 5 min is decreased. Such improvement can be related to the enhanced interfacial interaction between matrix and filler upon the filler surface modification. Reduction of the hysteresis loss together with smaller stress relaxation implies that the PMDS composites containing NDs are suitable candidates for the energy harvesting application.

## 4. Conclusions

The efficient surface functionalization of nanodiamonds allows coupling of the NDs to the silicone rubber matrix and can reduce the material-related losses of the composites. Carboxylated nanodiamonds were successfully modified with modifying agents containing epoxy-groups. The reaction between carboxylic and epoxy-groups at 40 °C offered a relatively easy way to decorate the ND surface with groups that can improve the interaction between ND and silicone matrix. In the case of 1,2-epoxy-9-decene containing vinyl-group ND can participate in the crosslinking process of the matrix, thus creating strong chemical interactions or in case of epoxy-compound containing silane an alkoxysilane group can be used for further surface modification. A direct reaction between carboxyl and silanol groups, however, was shown to be insufficient but due to the condensation of silanol groups a thick shell seems to be created around the ND clusters thus changing its surface. 

Surface modification of NDs improved the dielectric properties of ND-silicone composites. Only 0.1 phr of NDs is enough to reduce the dielectric loss of silicones mostly due to the lower contribution from the DC-conductivity. The addition of higher amounts of NDs does not improve the properties further. Among the different modified NDs, the treatment with 1,2-epoxy-9-decene reduces the dielectric losses at 1 Hz fivefold compared to the reference, but the effect deteriorates with increasing time owing to the change in the loss properties of the matrix. The same compound also led to about twice-decreased hysteresis loss at 10th cycle and a meaningful reduction of the stress relaxation. Therefore, the application of small amounts of NDs, especially those modified with 1,2-epoxy-9-decene, can be considered beneficial to improve the efficiency of silicones applied in dielectric energy generators.

## Figures and Tables

**Table 1:** Oxygen-containing groups on nanodiamond (ND) surface determined by Boehm titration method.

	Surface Groups, mmol/g			
	Carboxyl	Lactone	Phenolic	All Surface Groups
ND reference	0.131	0.101	0.219	0.451
ND oxidized	0.329	0.018	0.127	0.475

**Table 2:** Energy dispersive X-ray spectroscopy detector (EDS) analysis of filler particles.

Sample	Element Content, %			
	C	O	Si	Others
ND-COOH	96.0	3.2	-	<1
ND-VTMS	54.6	18.0	27.3	<1
ND-ED(40C)	96.0	4.0	-	-
ND-GOPTMS	90.8	6.6	2.6	-

**Table 3:** Moduli and apparent crosslink densities of silicone-ND composites (0.1 phr) and reference material.

Sample	Modulus, MPa			Apparent Crosslink Density 1/Q, -
	10%	50%	100%	
PDMS/0	0.12 ± 0.01	0.49 ± 0.03	1.34 ± 0.11	0.689 ± 0.005
PDMS_oil/0	0.12 ± 0.01	0.45 ± 0.01	1.38 ± 0.09	0.614 ± 0.003
PDMS_oil/COOH_0.1	0.11 ± 0.01	0.49 ± <0.01	1.13 ± 0.22	0.683 ± 0.003
PDMS_oil/VTMS_0.1	0.10 ± 0.01	0.47 ± 0.01	1.39 ± 0.02	0.674 ± 0.004
PDMS_oil/ED_0.1	0.10 ± 0.02	0.44 ± 0.01	1.38 ± 0.10	0.679 ± 0.003
PDMS_oil/GOPTMS_0.1	0.11 ± 0.01	0.45 ± 0.01	1.39 ± 0.03	0.554 ± 0.047

**Table 4:** Glass transition temperature and crystallinity of silicone compounds measured by differential scanning calorimetry (DSC).

Sample	*T*_g_, °C	Enthalpy, J/g		Degree of Crystallinity*, %
		Cold Crystallization	Melting	
PDMS/0	−127.0	3.26	3.84	0.9
PDMS_oil/0	−127.3	0.78	0.92	0.2
PDMS_oil/COOH_0.1	−124.8	1.45	15.37	22.7
PDMS_oil/VTMS_0.1	−120.6	-	15.47	25.2
PDMS_oil/ED_0.1	−121.6	-	14.90	24.3
PDMS_oil/GOPTMS_0.1	−126.6	9.57	13.58	6.5

* Based on the enthalpy of fusion of 100% crystalline PDMS = 61.3 J/g [46].

**Table 5:** Hysteresis loss and stress relaxation of silicone compounds.

Sample	Hysteresis Loss, %		Stress Loss, %		
	1st Cycle	10th Cycle	after 1 min	after 5 min	after 1 h
PDMS/0	1.49 ± 0.17	0.63 ± 0.20	1.08 ± 0.04	1.77 ± 0.11	2.83 ± 0.47
PDMS_oil/0	2.21 ± 0.27	0.64 ± 0.15	0.78 ± 0.13	1.30 ± 0.28	2.26 ± 0.38
PDMS_oil/COOH_0.1	1.70 ± 0.25	0.64 ± 0.37	0.61 ± 0.17	0.96 ± 0.23	1.98 ± 0.38
PDMS_oil/VTMS_0.1	1.59 ± 0.66	0.46 ± 0.23	0.56 ± 0.09	0.86 ± 0.06	1.47 ± 0.16
PDMS_oil/ED_0.1	1.89 ± 0.71	0.30 ± 0.10	0.69 ± 0.04	1.22 ± 0.28	2.50 ± 0.41
PDMS_oil/GOPTMS_0.1	1.48 ± 0.37	0.37 ± 0.18	0.62 ± 0.12	1.07 ± 0.21	2.41 ± 0.37

## Supplementary Materials

The following are available online at https://www.mdpi.com/2073-4360/11/7/1104/s1, Table S1: Properties of carboxylated NDs, Figure S1: Chemical structure of the surface modifiers used in the study, Figure S2: FTIR-ATR spectra of the mono-silanol group modified NDs, Figure S3: TGA mass loss and mass loss rate curves for the mono-silanol group modified NDs, Figure S4: DSC curves of PDMS-ND compounds.
