# Supplementary material for: Influence of Surface Modified Nanodiamonds on Dielectric and Mechanical Properties of Silicone Composites
Source: Polymers (Basel). 2019 Jun 29;11(7):1104. doi: 10.3390/polym11071104 (PMC6681107; doi:10.3390/polym11071104)
Supplement: Supplementary file 1 [file polymers-11-01104-s001.pdf]

Influence of surface modified nanodiamonds on dielectric and mechanical properties of silicone composites

Alexandra Shakun [1,*], Rafal Anyszka [2,3], Essi Sarlin[1], Anke Blume [2] and Jyrki Vuorinen [1]

[1] Tampere University, Materials Science and Environmental Engineering, P.O.Box 589, FI-33014 Tampere, Finland
[2] University of Twente, Elastomer Technology and Engineering, P.O. Box 217, 7500 AE Enschede, The Netherlands
[3] Lodz University of Technology, Institute of Polymer and Dye Technology, Stefanowskiego 12/16, 90-924 Lodz, Poland
* Correspondence: alexandra.shakun@tuni.fi;

Supplementary information

The properties of carboxylated NDs are presented in Table S1.

Table S1. Properties of carboxylated NDs*.

Property	Value
Single particle crystal size	4.2 ± 0.5 nm
Bulk density	~ 0.5 g/cm^3
Pycnometric density	3.1 - 3.2 g/cm^3
Specific surface area (BET)	330 m^2/g
Aggregate particle size distribution width (D_{10}/D_{50}/D_{90})**	~ 0.7/2.6/7.6 μm
Moisture content	≤2%
Nanodiamond content	≥ 97 wt. %
Oxidizable carbon content	≤ 2 wt. %
Incombustible impurity content	≤ 2 wt. %

*Information provided by the supplier

** D_{10}/D_{50}/D_{90} – 10%/50%/90% of the particles lies below this value

In order to address the probability of the reaction between carboxylic group of nanodiamond (ND) and the silanol groups, additional surface modifications were performed with single alkoxy group silanes. All compounds used for the study are presented in Figure S1. Vinyldimethylethoxysilane (VDMES) and octadecyldimethylmethoxysilane (ODMMS) purchased from abcr GmbH were used for ND surface modification following the same procedure as for vinyltrimethoxysilane (VTMS) used in the study. However, in case of mono-silanol group compounds, to maintain the same conditions of minimizing the condensation rate, the acid was not added. As seen from the Figure S1, these silanes were similar in structure except for the amount of alkoxy groups attached to the silicon atom.

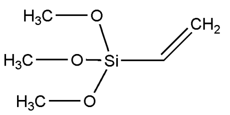

Vinyltrimethoxysilane

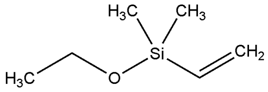

Vinyldimethylethoxysilane

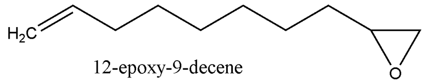

12-epoxy-9-decene

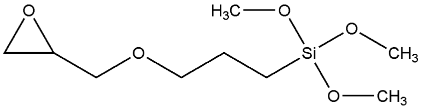

3-glycidoxypropyltrimethoxysilane

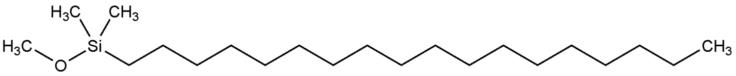

Methoxy(dimethyl)octadecylsilane

Figure S1. Chemical structure of the surface modifiers used in the study.

The modified samples were studied with FTIR-ATR. The resulting spectra are shown in Figure S2. For both modified samples, an increase in all peak intensities was noticed. In ND-VDMES sample, the peak at 1620 cm^{-1} corresponding to the presence of –OH group is especially pronounced. In ND-ODMMS, the peak shifts to 1624 cm^{-1}, which, together with an increased broad peak at 3400 cm^{-1}, is indicative of the presence of water or solvent. The presence of solvent is confirmed in Figure S3. Finally, in ND-ODMMS sample, the peaks at 2944, 2875 and 1455 cm^{-1} corresponding to $–CH_2–$, and 1745 cm^{-1} corresponding to C=O group of lactones become more apparent. Such changed are most probably related to the physical interaction between the surface oxygen containing groups of ND and adsorbed water.

For the ND-VDMES, no vinyl group bands around 3080/2997 cm^{-1} and 909 cm^{-1} could be seen, and the spectra looked very similar to the untreated ND-COOH. However, the described vinyl-related peaks may have been too low to be detected, and the spectra contained small peaks that did not appear in the untreated material, which are marked with arrows. Therefore, EDS and TGA analysis of the sample was performed. According to the EDS analysis, ND-VDMES sample contained 95.7% C and 4.3% O with traces of Al, which was very similar to ND-COOH and no presence of Si was detected. As seen from the Figure S3, no mass loss was detected at the temperatures around 200-300 °C, where attached organic molecules should decompose. However, unlike ND-COOH, the sample contained about 1% of adsorbed water or solvent.

For the successfully modified ND-ODMMS, a sharp and intensive characteristic band for a long aliphatic chain would appear at 720-725 cm^{-1}. However, the ND-ODMMS sample showed no such a peak and was similar to ND-COOH and ND-VDMES, as seen from Figure S1. No Si-C or Si-O peaks was noticed. The TGA data shown in Figure S3 was very similar to ND-COOH, and no degradation peaks were seen in the DTA curves at the temperatures below 500 °C. According to these test results, the reaction between the alkoxy and carboxylic group was considered improbable in such conditions.

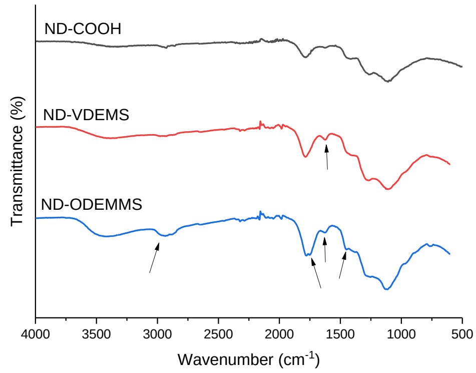

Figure S2. FTIR-ATR spectra of the mono-silanol group modified NDs. Differences in spectra are marked with arrows.

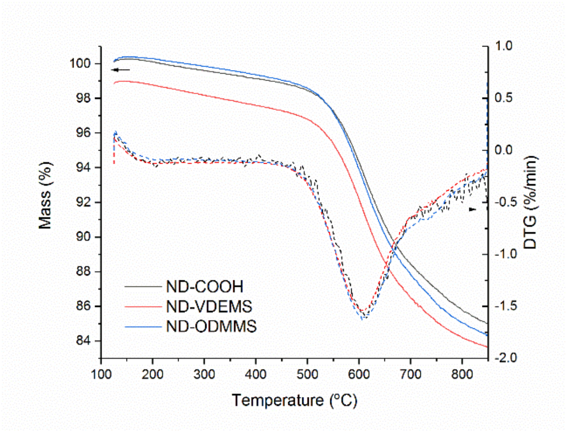

Figure S3. TGA mass loss and mass loss rate curves for the mono-silanol group modified NDs.

The DSC curves are presented in Figure S4. Significant melting peaks are seen for all compounds containing NDs.

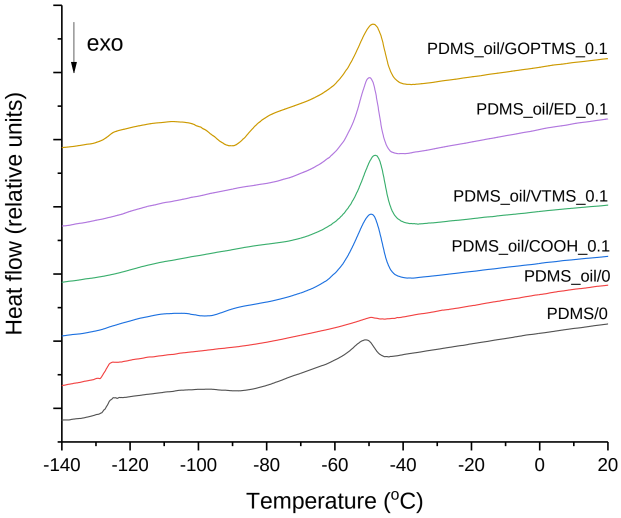

Figure S4. DSC curves of PDMS-ND compounds. The curves are shifted vertically for clarity.
